# Arsenical Poisoning

**Published:** 1879-11

**Authors:** 


### Editorial.

### ARSENICAL POISONING.

On the 27th of September, Mr. Geo. A. Gardner, of Brooklyn, N. Y., died, from disease of the mouth, neck and throat, induced, it is alleged, by arsenic, applied to destroy the "nerve" (pulp) of a tooth, by a dentist.

The first public statement of this case, so far as now appears, was made in the "*New York Times*" of October 6th, in which is the following:

"Mr. Geo. A. Gardner, nephew by marriage of Prescott, the historian, died in Brooklyn on the 27th ult., in great agony, after ten weeks of indescribable suffering. It is said by his attending physician that his death was caused by arsenical poison, placed by a dentist in one of his teeth for the purpose of killing an aching nerve."

"The certificate of death, which was filed with the department of health of Brooklyn, by Dr. Samuel S. Guy, of No. 302 Clinton street, states that the cause of death was gangrene of the mouth and face, arising from treatment of a tooth."

The great point of interest, to the dental profession at least, that attaches to this case hinges upon the oft-repeated statement that, the death was caused by the application of arsenic, by a dentist, to destroy the "nerve" of a tooth.

Now what are the facts in the case?

From the mass of confused and conflicting statements, there is credible evidence that Mr. Gardner died, and that he died from a remarkable and very severe affection of the mouth, throat and neck, induced most probably by a diseased tooth, or disease about tooth.

It is alleged that at the beginning, or at least in the early stage of this difficulty, the tooth involved, had received treatment at the hands of at least two dentists. Exactly what was done by either can only be known by their respective statements. These they have given, and they are such, as to carry conviction of their correctness to any unbiased mind; and when in connection with this, the professional character, integrity and social standing of both these gentlemen are taken into account, the statements made by certain persons, and the excitement arising from these unproved charges, are remarkable, to say the least.

Now what are the charges, by whom, and when made, and whom do they effect?

Prior to the death of Mr. Gardner, we hear of no charge of malpractice, in any respect, against Drs. Waters or Marvin, the two dentists who had treated the tooth.

That arsenic was the cause of Mr. Gardner's death, did not seem to have been entertained by any one, for some time, probably several days after his interment, for the certificate of death filed with the department of health of Brooklyn, by Dr. Samuel S. Guy, states that the cause of death was "gangrene of the mouth and face, arising from treatment of a tooth."

Had the thought of arsenical poisoning in this case, entered the mind of Dr. Guy before, or at the time of making the certificate of death, the omission of the statement of that fact, would have been to say the least criminal neglect.

It seems the "*Times*" reporter was the first one to agitate the subject. This was several days after the interment. Dr. Wyckoff, of the Brooklyn health department, informed the reporter that, from the high standing of Dr. Guy, and from the nature of his certificate, he decided that Mr. Gardner's death had been due to natural causes, and paid no further attention to it.

He had never known of a well established case in which death was directly and exclusively due to arsenical poisoning, which had been the result of a dental operation.

The first we have of Dr. Guy's charge of arsenical poisoning, is to the "*Times*" reporter during his investigation, to whom Dr. Guy said, "Mr. Gardner did die from the effects of arsenical poison, introduced into his system as a result of the treatment of his tooth, and there isn't the slightest doubt about it; it is a clear case.

The proper medical statement of the cause of his death, is that he died of septicæmia, following gangrene of the mouth and face caused by arsenic, placed in a tooth during treatment."

How did Dr. Guy know this?

And if this was the proper medical statement of the cause of Mr. Gardner's death, why was it not embodied in the certificate of Dr. Guy to the health officer? Is it possible that a case, so terrible in all its aspects, as this doubtless was, should have been due, in its beginning, in its awful continuance and its fatal result to arsenical poisoning, and yet this fact not be mentioned in the certificate of death? This should have been done irrespective of the method of the administration of the arsenic. Much more should it have been done if it had been administered through criminal carelessness.

Dr. Guy further states, "I did not see Mr. Gardner until the 15th of September, four days after he was attacked. The poison had then been actually absorbed."

A very remarkable statement indeed, for one to make, who did not know whether arsenic had been used at all or not, and how very searching his investigation must have been, to enable him to recognize and know, four days after the administration, "that the poison had been actually absorbed."

It would be very interesting indeed, if Dr. Guy would inform us, how this absorption took place. Was it through the canal of the tooth? Was it through the dentine and cementum? Or did it escape in part or in whole from the cavity into the mouth, and there dissolve in the saliva—it could not be absorbed till dissolved—arsenic is very sparingly soluble in saliva. Or did this imaginary fortieth to a sixtieth of a grain of arsenic pass into the stomach, and enter the system through that channel, enter the circulation and perpetrate its awful ravages upon the mouth and neck of the victim as described? What nonsense.

Those having any familiarity with arsenic poisoning, know that the symptoms are "faintness, depression, nausea and sickness, with an intense burning pain, in the region of the stomach, increased by pressure; the pain in the abdomen becomes more and more severe, and there is violent vomiting of a brown turbid matter, mixed with mucus, and sometimes streaked with blood.

These symptoms are followed by purging, which is more or less violent, and this is accompanied by severe cramps in the calves of the legs.

The vomited matters are in some cases covered with blood, and the mixture of blood with bile, has often given to them a green or brown color. There is a sense of constriction, with a feeling of burning heat in the throat, commonly accompanied by the most intense thirst.

The pulse is small, very frequent and irregular; sometimes wholly imperceptible.

The skin is cold and clammy, in stage of collapse; at other times it is very hot. The respiration is painful from the tender state of the stomach."

The stomach is invariably the seat of attack from arsenical poisoning.

Is it not rather strange that the leading symptoms of arsenical poisoning did not occur in this case, or were overlooked, or were not thought worthy of mention?

Now what is said as to the quantity of arsenic required to destroy life? Taylor says, the smallest fatal dose hitherto recorded was observed in a case communicated by Dr. Castle, of Leeds, to the *Provincial Journal* June 28th, 1848, page 347.

A woman took half an ounce of Fowler's solution (arsenite of potash) in unknown doses, during a period of five days. She then died, and on examination, the stomach and intestines were found inflamed. Death took place by syncope, (mortal fainting) and there was an absence of vomiting and purging.

The quantity of arsenic which here destroyed life could not have been more than two grains.

In another case two grains and a half of arsenic contained in two ounces of fly water, killed a robust healthy girl, aged nineteen, in thirty-six hours. Hence under circumstances favorable to the operation of the poison, the fatal dose in an adult, may be assigned at from two to thiee grains."

The authority here quoted places the oft repeated statement of Dr. Guy, "that this was a clear case of arsenical poisoning," in a very unenviable light at the best, and especially so, since he adduces no evidence that arsenic was used at all.

It may not be amiss here to note the statements, made by Dr. Waters, of Boston, and Marvin, of Brooklyn, the only dentists so far as known, who gave any treatment to Mr. Gardner's tooth.

Dr. Waters says. "That he did not use arsenic in Mr. Gardner's tooth, but simply a solution of carbolic acid," the aim and tendency of which was to disinfect and allay irritation. He says he has not used arsenic in dental practice for twenty years.

Dr. Marvin says, "As to the case of Mr. Gardner, I know very little about it, having seen the gentleman but once, and then only for three or four minutes. He called with an aching tooth, saying that his Boston dentist had been treating it; that he was obliged to come to New York on business; that it had pained him more or less all night. I found it temporarily filled, removed the temporary filling, made an application of creosote, hoping thereby to give him relief from pain, till he could return to his dentist, which I recommended him to do. He then left, and I saw him no more."

Now if these gentlemen are credible witnesses, who applied arsenic?

In the newspaper reports, comments and interviews in reference to this case, many wild, reckless and incorrect statements have been made. Some of which, it is to be hoped, for the reputation of the interviewed, have been very much distorted, in the passage from the lips to the printing press.

The aim of this criticism is simply, to correct any false impression that may be made by the faulty and reckless manner of presentation of this case to the public.

It is in no sense, intended as a defense or an encouragement, of the very common practice of using arsenic for destroying the pulps of teeth, a practice which I regard as reprehensible in almost every case, not however from danger of poisoning, for in no case is the amount required for destroying the pulp of a tooth or twenty teeth, enough to make even the slightest impression, in the way of systemic poisoning; but because of the indiscriminate ruin wrought upon the teeth with it. There are probably a thousand pulps destroyed, when not more than one should be. It would probably be better for the profession and for the people, if it was wholly discarded by dentists for this purpose. With the present ability and resources for the treatment and management of exposed pulps, arsenic is not needed.

